# The Medical Department of the University of Michigan

**Published:** 1855-05

**Authors:** 


### *The Medical Department of the University of Michigan.*

In the March number of our Journal we informed its readers that the Legislature of Michigan had passed a law requiring the establishment of a Chair of Homœopathy in the medical department of their University at Ann Arbor. In the last number of the Peninsular Journal (the organ of that school) the fact is fully admitted; but the Editor labors through several pages to show that the act of the Legislature has no legal force, the University in all its departments being placed, by the Constitution of the State, exclusively under the control of a Board of Regents, whose powers and duties are independent of Legislative instructions.

We hope, for the credit of the State and of the profession, that these views are correct, and that the Board of Regents will exhibit more good sense than the recent Legislature. And yet our friends at Ann Arbor cannot feel very secure while they remember that the Regents themselves are elected by the same people who elected the Legislature. All experience has shown that the assumption of a position antagonistic to the mandates of a popular Legislature, though capable of postponing certain results, is generally more disastrous in the end than ready obedience. The cry of monopoly, the charge of shrinking from free discussion and an *equal* participation in the benefits of the State University, and the vulgar accusations of illiberality and old fogyism, wielded by demagogues and interested partizans, will rally an opposition which the Faculty at Ann Arbor, even if aided by the unanimous action of the Regents, will find it no easy task to withstand. In this contest the Faculty have our sincere sympathy. We notice, however, in the same number of the Peninsular Journal, a somewhat lengthy review of Dr. Cabell's Report on Medical education, contained in the last volume of the Transactions of the American Medical Association, the closing paragraph of which very complacently claims that the medical department of their University is admitted to be "far in advance" of other medical schools in their system of education, requisitions, &c. The only foundation we could find for this arrogant flourish was a quotation which Dr. Cabell had made from a previous report of Dr. Pitcher, of Detroit, to the effect that it was "in contemplation to adopt the same standard of qualifications, in regard to preliminary education, for admission of students to the medical as to the literary department of the University." Now, if we recollect aright, this same thing was said to be in contemplation in one of the first circulars issued in reference to the organization of that school. It is still, we presume, "in contemplation" Meanwhile the Faculty are annually admitting students without the slightest reference to preliminary qualifications of any kind. How long they will continue their "contemplations" on the subject, adroitly publishing it just before each annual meeting of the National Association, as a matter just about to assume great practical importance, we do not know. But we do know that such baseless pretensions indicate a very weak spot somewhere.

